# Self-Evaluation Strategies in College Women Trying to Lose Weight: The Relative Use of Objective and Social Comparison Information

**DOI:** 10.3389/fpsyg.2020.01254

**Published:** 2020-06-10

**Authors:** Heidi A. Wayment, Brian A. Eiler, Keragan Cavolo

**Affiliations:** ^1^Department of Psychological Sciences, Northern Arizona University, Flagstaff, AZ, United States; ^2^Department of Psychology, Davidson College, Davidson, NC, United States

**Keywords:** self-evaluation standards, self-evaluation motives, social comparison processes, weight loss, college females

## Abstract

We examined patterns of self-evaluative information use in a sample of college women who were trying to lose weight (*N* = 306). Participants described their weight loss experiences and answered questions about their self-evaluative activity via an online survey. The analysis strategy examined the relative use of four types of self-evaluative information (objective, upward social comparison, lateral social comparison, and downward social comparison) to meet three basic self-evaluative motives (accurate self-assessment, self-enhancement, and self-improvement). We also examined the role that dissatisfaction, uncertainty, importance, and self-esteem played in the relative use of information and the relationship of these factors on weight loss success. Our findings support previous research showing the primacy of accurate and self-improvement motives in the domain of weight loss and the usefulness of lateral social comparison information for meeting all three motives. Women evaluating their weight reported using upward social comparison information most often, followed by objective information. Lateral and upward social comparison information were rated as more useful than downward social comparison information for meeting accuracy and self-improvement motives. Both lateral and downward social comparison information were reported as especially useful for self-enhancement, with upward social comparison information rated as least useful. Our study utilized an integrative approach for understanding self-evaluative processes in the area of college women’s weight loss. We found general support for our hypotheses regarding well-documented patterns of social comparison information usefulness for meeting three self-evaluative motives. Our data also support earlier research arguing that it is important to view information use in the context of multiple self-evaluative motives.

## Introduction

Self-evaluation, the process by which individuals seek information to assess their own performance in a domain, has a rich scientific history (Festinger, 1954; Albert, 1977; Wills, 1981; Wood, 1989; Sedikides and Strube, 1997). Research has focused on the different motives that self-evaluation processes serve, including accurate self-evaluation (i.e., objective accounts of performance, skills, and traits that enable individuals to anticipate and control their future behavior; Festinger, 1954; Schachter, 1959; Trope, 1975; Swann, 1983), self-enhancement (i.e., the desire to protect a sense of self-worth in the face of threat; Wills, 1981; Taylor and Brown, 1988; Tesser, 1988), and self-improvement (i.e., extracting information that is useful for bettering one’s situation and guiding future behavior; Markus and Nurius, 1986; Taylor and Lobel, 1989). In addition, a great deal of attention has been devoted to the types of information people gather about themselves or others to pursue personal self-evaluation needs such as, objective (Festinger, 1954), social comparison (Festinger, 1954; Suls, 1977; Wills, 1981), or temporal comparison information (Albert, 1977). Other researchers have focused on moderating conditions that may influence the motive or the type of information guiding self-evaluation, such as threat, dissatisfaction, uncertainty, control, and/or the importance of a self-domain. Self-esteem is also an important moderator of information use, with individuals high in self-esteem more able to extract self-enhancing information and avoiding unflattering comparisons (Wayment and Taylor, 1995). It has also been observed that individuals low in self-esteem have less stable self-concepts and may be more influenced by social comparison information (Campbell, 1990). Wills (1981) argued that low self-esteem can make self-enhancement motives more prominent.

All of these dimensions were incorporated into an integrative model of self-evaluation processes (Wayment and Taylor, 1995, see also Helgeson and Mickelson, 1995). Collectively, studies utilizing this model have shown some general preferences for self-evaluative information given the domain under evaluation and self-evaluative motive.

This paper utilized this integrative model to examine the self-evaluative information college women use to assess their weight loss. We chose this domain because of its relevance to college-aged women (Wharton et al., 2008) and the widespread availability of objective information in this domain. Further, objective information is especially important in this domain because of its usefulness for accurate self-evaluation, which is associated with weight loss success (Wharton et al., 2008; Riggs et al., 2017). However, accurate self-evaluations are but one important self-evaluative motive. To date, no studies have simultaneously examined the types of self-evaluative information that are perceived as useful for meeting all three self-evaluative motives in women who are trying to lose weight. For example, well-executed studies of the affective consequences associated with exposure to upward social comparison information (media images, in-person comparisons) support the idea that upward social comparison information is not very useful for self-enhancement (Tiggemann and McGill, 2004; Fardouly et al., 2017). Yet, other information sources have not been examined for their potential usefulness for meeting accuracy and self-improvement motives.

In the following sections, we describe general patterns of results using the integrative model in other domains, describe the relevance of examining self-evaluation patterns in college women seeking to lose weight, and describe our hypotheses (see Halliwell and Dittmar, 2005 regarding the importance of distinguishing between accuracy and self-enhancement motives in domain of body image evaluation).

### Self-Evaluation Strategies and Weight Management

Excessive weight contributes to higher risk for diabetes, coronary heart disease, various cancers, and sleep problems, and has even been considered a global epidemic (Calle et al., 1999; Must et al., 1999; Kopelman, 2000). The saliency of weight and weight loss is especially prevalent for college-aged women. For example, female college freshmen gain ~5.5 lbs. over the first year, and this weight gain relates to lower academic confidence and changes in healthy eating (Economos et al., 2008). Moreover, disproportionate attention is paid to weight in college regardless of whether one’s weight is considered objectively healthy or unhealthy (Wharton et al., 2008). It also seems that college-aged women are more concerned with losing weight, as female freshman and sophomores are more likely than their male counterparts to be actively trying to lose weight despite lower overall levels of obesity (Lowry et al., 2000). Finally, given that early college experience is a known risk factor for weight gain (Vella-Zarb and Frank, 2009), it is important to identify constraints (e.g., self-evaluation) on weight management (e.g., weight loss) for college-aged women.

Some research has suggested that self-evaluation strategies may impact weight loss success. For example, social comparison information use has been associated with body dissatisfaction and dieting (Engeln-Maddox, 2005; Myers et al., 2012; Shakya et al., 2015). In one study, those who perceived themselves to be similar to a prototypically overweight person were more likely to diet for weight loss (Dalley and Buunk, 2009, 2011). Relatedly, Lewallen and Behm-Morawitz (2016) demonstrated that upward social comparison may be associated with intentions to engage in extreme weight loss behaviors. Further, naturalistic studies addressing self-evaluation preferences regarding women’s body image have shown that women are more likely to make upward social comparisons than lateral or downward social comparisons when comparing their body to others (Myers et al., 2012; Fardouly et al., 2017; Betz et al., 2019). These studies link the specific use of social comparison information with appearance, weight, and dieting outcomes, but do not isolate how specific types of self-evaluative information are useful for meeting all three self-evaluative motives.

Finally, women’s perceptions of weight and body image is strongly linked to self-esteem issues and has been implicated in the negative impacts associated with media exposure (Vogel et al., 2014). Low self-esteem has been associated with greater self-evaluation activity (Wayment and Taylor, 1995). In their study of women’s disordered eating, Tylka and Sabik (2010) found that women with low self-esteem were more likely to engage in social comparison activity and utilize unrealistic standards for body weight. Thus, in the area of women’s weight loss, it is important to consider moderating variables that can influence self-evaluative activity.

### Current Study

The research presented here focuses^1^ on the relative use of objective standards and social comparison information and their perceived usefulness to meet three self-evaluative motives in the domain of college women’s weight loss. Our hypotheses were based on the integrative model of self-evaluation (Wayment and Taylor, 1995)^2^ . Given the ubiquity of objective information in the area of weight and weight loss, we first expected that college women would report using objective information most often (H1), and of the three types of social comparison information, we expected a greater use of upward social comparison information (H1a). We had two competing hypotheses regarding the relative use of objective and social comparison information types. Festinger’s (1954) original formulation of social comparison theory argued that people prefer objective information, but when unavailable, would turn to social comparison information to meet their self-evaluative needs. Bandura (1982) argued that objective information should increase the use of social comparison information to refine its meaning. Thus, we examined the relative use of objective and social comparison information for evaluating weight loss (R1).

We also ventured several self-evaluation motive-by-information use hypotheses. First, for accurate self-evaluation, objective information was expected to be perceived as most useful, and more useful than all three types of social comparison information (H2). Of the three social comparison information subtypes, lateral social comparison information was hypothesized to be perceived as the most useful for meeting accuracy goals, and more useful than upward or downward social comparisons (H2a). Objective information was also expected to be perceived as most useful to meet the self-improvement motive (H3), followed by upward social comparison information (H3a). Finally, we predicted that objective information would be perceived as useful for self-enhancement (H4) and downward social comparison information would be most useful to meet the self-enhancement motive, and more useful than upward or lateral social comparisons (H4a). We examined two additional research questions. First, how are moderators of information use (*importance* of weight loss, *uncertainty* about weight loss progress, *amount of control* over one’s weight loss, *dissatisfaction* with weight loss, self-esteem) related to frequency of information use? (R2). Second, are specific types of self-evaluative information use associated with perceptions of weight loss success? (R3).

## Materials and Methods

### Participant Recruitment and Procedure

The current study recruited Introductory Psychology students who were trying to lose weight. A total of 357 female students completed an online survey and were compensated with partial course credit. To establish the final sample, several participant features were examined for exclusion. Participants were excluded for evidence of “satisficing” (*n* = 51)^2^, identified as male (*n* = 46), were age outliers (i.e., > 3 *SD* older than the mean, *n* = 2), or identified as transgender (*n* = 1) or genderqueer (*n* = 1). The final sample (*N* = 306) was entirely female, had an average age of about 18 (*M* = 18.38, *SD* = 0.77), and were predominantly White (68.3%). The racial demographics of the rest of the sample were: Hispanic (17.3%), African American (6.5%), Asian (4.6%), and American Indian (4.6%).

### Measures

#### Demographics

Participants provided their age, gender, level of education, and race.

#### Weight Loss Goals

Participants were asked open-ended questions regarding their weight loss goals, the reason(s) they were trying to lose weight (via a forced choice question including: fitness, appearance, health, attractivity, and other), and were instructed to write an open-ended response discussing their personal experiences with their “weight loss journey” as if writing on a blog (e.g., Reddit) for others who were also interested in weight loss to see.

#### Weight Loss Success

A single item assessed participants’ perception of their weight loss success (1 = not at all successful; 6 = very successful).

#### Length of Time Pursuing Weight Loss

Participants indicated how many months they had been trying to lose weight.

#### Body Size Perceptions

Participants rated their current and ideal body size by marking along a 0–100 point continuum anchored by two graphic images (of a female body) at each endpoint (Gardner et al., 1998). A difference score (i.e., between current and ideal perceptions) was created such that larger values were indicative of greater current-ideal body size discrepancy.

#### Body Mass Index (BMI)

Participants were asked to provide their height and weight to calculate individual participant BMI scores (Centers for Disease Control and Prevention, 2014). Higher BMI values reflect greater body mass relative to height. For reference, a BMI below 18.5 is considered underweight, between 18.5 and 24.9 is considered normal/healthy weight, between 25.0 and 29.9 is considered overweight, and above 30.0 is considered obese.

#### Information Use Moderators

Four, one-item questions assessed potential situational correlates of information use: “How *important* is it for you to reach your current weight loss goal?” (1 = not very important; 7 = very important), “How *satisfied* are you with your weight loss progress so far?” (1 = very dissatisfied; 7 = very satisfied), “How much *control* do you feel that you have over reaching your weight loss goal?” (1 = very little control; 7 = a great deal of control), and “How *certain* are you that you will reach your current weight loss goal?” (1 = not at all certain; 7 = very certain). Mean scores for control and certainty were statistically similar (*M* = 4.50, *SD* = 1.57; *M* = 4.58, *SD* = 1.45, *t*_paired_ = -0.971, *p* = 0.33) and were highly correlated (*r*^2^ = 0.62, *p* < 0.001). As such, these two items were reversed and averaged into a single score called uncertainty (*M* = 2.46, *SD* = 1.35). The 10-item Rosenberg Self-Esteem Scale (Rosenberg, 1965) was used to measure global self-worth and included negative and positive self-related questions (e.g., “On the whole, I am satisfied with myself,” “I wish I could have more respect for myself-” *reversed*). Responses were recorded on a 5-point Likert-type scale, ranging from *strongly agree* to *strongly disagree*. Scores were reversed and summed, thus higher scores indicated higher self-esteem. The scale was reliable (α = 0.90) and the mean self-esteem score for this sample was around the midpoint of the scale’s range (*M* = 25.43, *SD* = 7.06).

#### Measures of Information Use and Usefulness

Participants answered questions regarding their use of 10 types of information. To align the hypotheses most closely with Festinger (1954), only objective, upward, lateral, and downward social comparison information were included in the analyses presented here. Questions were nearly identical to those used in Wayment’s (1992) original study. For each information type, participants were first provided with a definition and a brief example. The description of objective information included “For example, to evaluate one’s weight loss, an individual may seek out information about healthy weight from expert sources or weigh themselves on a scale or use other objective measures (body mass index, weight charts, etc.).” The description of upward social comparison information included “… may compare their weight with people who are doing better than they are. For example, they compare their weight with someone who weighs less than they do or someone who has been more successful in their weight loss.” The description of lateral social comparisons included “… may compare their weight with someone who weighs about the same as they do or someone who has had the same level of success/failure in their weight loss.” The description of downward social comparisons included “…may compare their weight with someone who weighs more than they do or someone who has been less successful in their weight loss.”

Following the provided examples, participants were given an opportunity to list examples of the information type in question the use to “evaluate their weight.” This open-ended question was followed by a question to assess frequency: “How often do you use [type of information] to assess your weight loss?” (1 = not at all, 7 = very frequently). Next, we asked three questions about the specific usefulness for each information type with respect to meeting self-evaluative motives: accuracy [How useful is (information type) for accurately evaluating your weight loss? (1 = not at all useful, 7 = very useful)], self-enhancement [When you evaluate your weight loss with (information type), how does it make you feel (1 = very bad, 7 = very good)], and self-improvement [How helpful is (information type) for improving your ability to lose weight? (1 = not at all helpful, 7 = very helpful)]. The order in which participants were asked to complete questions for each of the information types were presented randomly.

## Results

### Preliminary Analyses

Prior to conducting analyses, data were screened for outliers and missing data. A handful of items had missing respondents (<1% of sample) and mean replacement was used (Tabachnick and Fidell, 2013) to complete the data set. There were no violations of normality, as skewness and kurtosis values for all study measures fell within ± 2, indicating no extreme departures (Tabachnick and Fidell, 2013).

### Weight and Weight-Related Perceptions

On average, participants weighed about 148 pounds (*SD* = 31.43), had a weight loss goal of 17.67 pounds (*SD* = 18.84), and had been trying to lose weight for 4.58 months (*SD* = 8.72; *Range* = 72 months). Scores on the body size perception scale averaged 45.92 (*SD* = 22.31), which equated to approximately halfway between the thin and the obese drawings on either scale endpoint. The “ideal” average was 25.53 (*SD* = 15.83), with the average discrepancy between these two perceptions equaling 20.89 (*SD* = 13.62) on a 100-point scale. Most BMI scores (65%) were in the “normal” range with respect to Centers for Disease Control and Prevention (2016) standards (*M* = 24.85, *SD* = 4.91, *range* = 17.94–50.29). One participant was “underweight” (BMI < 18%), 24.6% were “overweight” (BMI = 25–30%) and 10.5% were in the “obese” range (BMI > 30%). BMI was positively correlated with the body size perception scale (*r*^2^ = 0.69, *p* < 0.0001). Respondents’ reasons for wanting to lose weight spanned fitness (75.5%), appearance (79.7%), health (68%), and attractiveness (52%). The average score for weight loss success was 4.14 (*SD* = 1.19) on a 6-point scale. The distribution of responses on this scale were as follows: 10.2% rated their success as 1 or 2, 42.8% rated their success a 3 or 4, and 47% rated their success as a 5 or 6.

### Frequency of Information Use

The first hypothesis was only partially supported. As expected, objective information was reported as used very often, but respondents reported using upward social comparison even more. Table 1 displays the average frequency ratings for each information type. Upward social comparison information was used most often, followed by objective information, and finally, lateral social comparison information. Downward social comparison information was used least frequently. More frequent use of objective information was positively associated with more frequent use of lateral social comparison information, modestly correlated with more frequent use of upward social comparison information, and unrelated to the frequency of downward social comparison information use.

**TABLE 1:** Product moments and correlations among information use frequency (*N* = 306).

	***Mean***	***SD***	**Skewness**	**Kurtosis**	**Downward**	**Lateral**	**Upward**
Objective	5.04	1.64	−0.664	−0.107	0.101	0.213***	0.130*
Downward	3.58	2.04	0.211	−1.19	–	0.225***	0.243***
Lateral	4.41	2.00	−0.326	−1.01		–	0.290***
Upward	5.28	1.65	−0.913	0.270			–

*Indicates p < 0.01, ***indicates p < 0.001; Usefulness of each information type was rated on a 7-point scale.

### Which Types of Information Are Most Useful for Meeting Self-Evaluative Motives?

**FIGURE 1:**
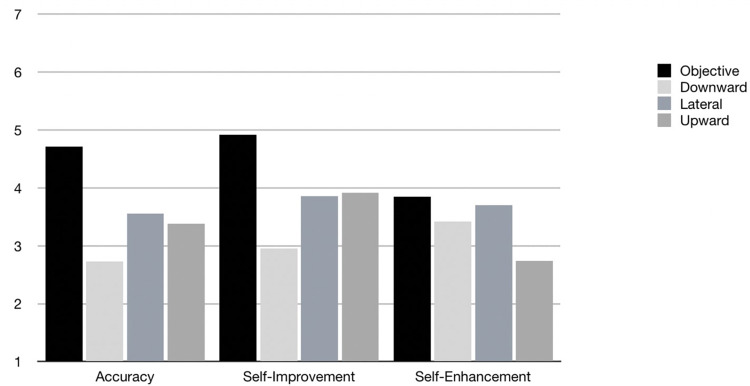
Relative perceived usefulness of information type for meeting each of three self-evaluative motives.

To test the remaining hypotheses, we computed a two-way (4 × 3) repeated measures analysis of variance with four levels of information use (objective, downward, lateral, and upward) and three levels of self-evaluative motives (accuracy, self-enhancement, and self-improvement). Information usefulness was the dependent variable. A Mauchly’s test revealed a violation of the sphericity assumption, therefore the Hyunh-Feldt estimates for *F* values and degrees of freedom were used in line with Field’s (2013) suggestion for sphericity estimates greater than 0.75. Planned contrasts were computed to examine specific predictions. The two-way interaction was significant, *F*(4.93, 1502.56) = 52.93, *p* < 0.0001 and interpreted with respect to the three hypotheses. The marginal means and 95% confidence intervals are presented in Table 2. For ease of interpretation, Figure 1 provides a summary of the perceived usefulness of all four information types for meeting the three self-evaluative motives.

**TABLE 2:** Mean usefulness of each information source compared across motives (*N* = 306).

	**Accuracy**			**Self-enhancement**			**Self-improvement**		
	**Mean**	***SE***	**95% CI**	**Mean**	***SE***	**95% CI**	**Mean**	***SE***	**95% CI**
Objective	4.71^1a^	0.07	[4.56, 4.86]	3.85^1b^	0.09	[3.67, 4.03]	4.92^1a^	0.08	[4.74, 5.09]
Downward	2.73^3b^	0.09	[2.55, 2.91]	3.42^2a^	0.09	[2.24, 3.61]	2.95^3b^	0.10	[2.76, 3.15]
Lateral	3.56^2a^	0.09	[3.37, 3.74]	3.70^1a^	0.09	[3.37, 3.74]	3.86^2a^	0.11	[3.65, 4.07]
Upward	3.38^2b^	0.10	[3.19, 3.56]	2.74^3c^	0.09	[2.56, 2.91]	3.21^2a^	0.10	[3.72, 4.12]

*For each information type (i.e., row), self-evaluative motive means not sharing a superscripted letter are significantly different (p < 0.001). For each motive (i.e., column), information type means not sharing a superscripted number are significantly different (p < 0.001). Please see Figure 1 for a visual depiction of these results.*

As predicted, objective information was perceived as both the most useful, and significantly more useful than any other type of social comparison information for accurate self-evaluations of weight loss (H2a). Both lateral and upward social comparison information were perceived as more useful for meeting accuracy goals than downward social comparison information (H2b). To meet self-improvement motives, we predicted that objective information would be preferred. This hypothesis (H3a) was strongly supported. Furthermore, we expected that upward social comparison information would be perceived as more useful than lateral or downward social comparisons to meet self-improvement goals (H3b). In partial support of H3b, both lateral and upward social comparison information were more useful than downward social comparison information. Regarding the types of information perceived as useful for self-enhancement, hypotheses were partially supported: objective and lateral social comparison information were perceived as most useful for self-enhancement goals, followed by downward social comparison information. As expected, upward social comparison information was perceived as the least useful information type for self-enhancement. In summary, objective information was rated as most useful for meeting accuracy and self-improvement goals. Upward social comparison information was perceived as useful for meeting self-improvement and accuracy motives. Lateral social comparison information was perceived as useful for all three motives. Downward social comparison information was perceived as most useful for meeting the self-enhancement motive.

### Correlations With Information Use

Correlations are reported in Table 3. Importance of weight loss was positively and significantly associated with more frequent use of objective, lateral social comparison, and upward social comparison information types. These types of information, as reported earlier, were especially useful for meeting accuracy and self-improvement motives. Dissatisfaction with one’s weight loss was associated with more frequent downward social comparison information use, which was noted as being useful for meeting the self-enhancement motive. Uncertainty with one’s weight loss progress was positively related to more frequent use of upward social comparison information. Finally, self-esteem was associated with less frequent use of upward social comparison information. Women who reported being more successful with their weight loss also reported being more satisfied with their weight, less uncertain about their weight loss progress, and had higher self-esteem. Finally, women who noted being more successful also described using less upward social comparison information.

**TABLE 3:** Correlations between information use frequency and moderating variables (*N* = 306).

**Information type**	**Importance**	**Dissatisfaction**	**Uncertainty**	**Self-esteem**	**Weight loss success**
Objective	0.22***	0.07	0.04	0.03	–0.02
Downward	0.04	0.14*	0.10	–0.04	–0.13
Lateral	0.12*	0.07	0.09	0.07	–0.03
Upward	0.18**	0.10^+^	0.16**	−0.14**	−0.16**
WL Success	–0.04	−0.56***	−0.50***	0.26***	–

^+^p < 0.07, *p < 0.05, **p < 0.01, ***p < 0.001. WL, Weight Loss.

## Discussion

This study examined the self-evaluative strategies female college students employed in their first semester of college, who also self-reported as “trying to lose weight.” The sample appears to be fairly typical of other female samples of college students used in weight loss research (Anderson et al., 2003; Herring et al., 2014; Vargas et al., 2014; Zeigler-Hill and Noser, 2015; Dakanalis et al., 2016). The women in this sample had BMIs that ranged from 17.94 (“underweight” according to Centers for Disease Control and Prevention, 2016) to 50.29 (“extreme obesity” according to the Centers for Disease Control and Prevention, 2016). The sample average was at the top end of what the CDC classifies as “normal” weight, with 24.6% in the “overweight” range and 10.5% in the obese range (Centers for Disease Control and Prevention, 2016). The BMI distribution in the current sample is similar to other studies (cf., Herring et al., 2014; Vargas et al., 2014; Dakanalis et al., 2016). On average, women reported trying to lose, on average, nearly 18 pounds, similar to what has been reported in a sample of college-age women (Anderson et al., 2003).

### Patterns of Self-Evaluation Activity

The results support the importance of two basic self-assessment motives, accuracy, and improvement, as most relevant for college women trying to lose weight (Festinger, 1954). Accordingly, upward social comparison information was reported as used most frequently, followed by objective information, to evaluate their weight loss goals and progress (Johnson and Stapel, 2010; Meier and Schäfer, 2018). Results supported Festinger’s (1954) original theory that, objective information, when available, is an extremely important and efficient way to establish an individual’s understanding of where they stand in a self-related domain. Objective information was also rated as the most useful information type for meeting self-improvement goals, a motive described as a natural consequence of having an accurate idea of one’s standing in any self-domain (Festinger, 1954). As expected, downward social comparison information was rated as used least often, and least useful for meeting accuracy and self-improvement goals, also supporting earlier research (Taylor and Lobel, 1989; Buunk et al., 1991).

Self-enhancement goals were perceived as being met best by comparisons with similar others (lateral social comparison information) and those not doing as well (downward social comparison). These results also provide continuing, albeit modest, support for the usefulness of downward social comparison information for meeting self-enhancement needs. Although reported as the least frequently used type of information to assess one’s weight loss progress, downward social comparison information was seen as most useful for meeting self-enhancement goals that for accuracy or improvement.

### Similar Others as Referents

We expected lateral social comparison information to be perceived as very useful for meeting accuracy goals, as Festinger (1954) originally theorized. In addition, we found compelling data supporting the idea that comparisons to similar others are also favored for meeting self-enhancement and self-improvement goals. For example, lateral social comparison information was rated by participants as more useful for meeting self-enhancement needs than comparing oneself to a worse performing other. Further, we found an especially strong relationship between objective information use and lateral social comparison information use. Although the mean ratings of the usefulness of information use from the current study closely parallel those reported by Wayment (1992), the results regarding lateral social comparison information stand out. Perhaps one reason why lateral social comparison information figured so prominently in this sample is the increase in social media use, specifically with respect to the information college-students receive about and from their friends. Given that friends are often perceived as self-similar, it is reasonable that information from “similarly performing others” might evoke comparisons largely comprised of friends. One additional interesting anecdote was that some participants mentioned it made them feel better to know they were not going through the process of losing weight on their own. Unfortunately, there are not many studies that compare the utility of comparing oneself to similar others for meeting self-enhancement motives since many studies only contrast use and preference for downward and upward social comparison information (e.g., Taylor and Lobel, 1989; Morganstern, 2007; Nabi and Keblusek, 2014). In one recent exception, Fardouly et al. (2017) found upward and downward comparisons were used more often than lateral comparisons for women evaluating their appearance. It could be argued that evaluating one’s “appearance” is a different self-domain than weight loss, thus further study into the relative use of lateral and upward social comparison with respect to weight loss is warranted.

### Moderators of Information Use Frequency

Two important components of Wayment and Taylor’s (1995) integrative model of self-evaluative processes are the situational and individual-difference influences on motive and information use preferences. The perceived importance of “weight loss goals” was positively correlated with frequency of objective information, upward social comparison, and lateral social comparison information use. This pattern supports previous findings that demonstrate self-evaluation activity as more likely to occur when the domain under evaluation is important (Wayment and Taylor, 1995). Dissatisfaction with one’s weight loss progress was significantly associated with increased use of downward social comparison information, also supporting earlier research (Wills, 1981). Greater uncertainty about one’s weight loss progress was associated with more frequent use of both upward, and downward, social comparison information. Lastly, self-esteem was negatively related to the frequency of upward social comparison information use. Overall, and consistent with research utilizing the integrative model (Wayment and Campbell, 2000; Gotwals and Wayment, 2002), self-evaluative activity was more frequent for those participants who believed their weight loss was important and for those who were dissatisfied and uncertain about their progress. That being said, the type of information they chose to use varied in perceived usefulness for meeting the three different self-evaluative motives.

### Self-Evaluation Strategies and Perceptions of Weight Loss Success

Finally, we examined the degree to which self-evaluative information use was associated with perceptions of weight loss success. Not surprisingly, individuals who were dissatisfied with their weight loss progress also reported less success. The perception of weight loss success was also correlated with self-esteem such that, compared to those lower in self-esteem, those with stronger self-esteem reported greater weight loss success. Participants who reported relatively less success were also more likely to say they were uncertain about their weight loss progress. Moreover, perceptions of weight loss success were unrelated to the perceived importance of weight loss. Perhaps the most interesting finding here was that the very information perceived as useful for self-improvement (and the type of information respondents said they used most often), upward social comparison information, was negatively related to success perceptions. That is, individuals who used relatively more upward social comparison information to assess their weight loss progress reported being less successful. Given the correlational nature of these data it is still unknown whether using upward social comparison reduces a person’s sense of success (i.e., upward social comparisons may not be particularly useful for meeting self-enhancement needs), or whether those who feel less successful seek out upward social comparison information (i.e., use this information to meet self-improvement needs). This is the conundrum associated with the use of upward social comparison – such information can be useful for self-improvement, but at the same time may pose a type of self-evaluative threat (Taylor and Lobel, 1989). It could also be that high self-esteem women are somehow able to engage cognitively with upward social comparison information in ways that not only buffer them from the potentially negative affect and instead inspire behavioral regulation (cf. Feeney et al., 2005). In support of this claim, Wayment’s (1992) earlier investigation of college students’ evaluation of their academic performance found that those high in self-esteem (compared to those low in self-esteem) found all types of information self-enhancing.

### Strengths and Limitations

One of the main strengths of the current study is that it was a conceptual replication of work conducted nearly 25 years ago by Wayment and Taylor (1995), who argued that multiple types of self-evaluative information and motives should be examined simultaneously (see also Helgeson and Mickelson, 1995; Cramer et al., 2016 for similar arguments). This study used identical item wording and scale endpoints to assess information use – albeit online instead of on paper. Thus, the results from the current study suggest that the integrative model and method to assess self-reported information appears to be as useful in 2019 as it was in 1992^3^. Another strength is that because respondents completed the survey online, we identified and removed participants who engaged in “satisficing” behavior (Barge and Gehlbach, 2012; Zhang, 2013).

The study also has several important limitations. A major limitation is that we used self-reported estimates of body weight, self-evaluative activity, and indicators of weight loss success. All of these measures are subject to responses constrained by social desirability concerns. We also could have asked additional questions or phrased them differently. For example, our question regarding perceived weight loss success did not have a specific time frame. Our rationale was that each participant had been on their weight loss journey for differing amounts of time (e.g., we asked participants how long they had been trying to lose weight and how satisfied they were with their weight loss “so far”). The absence of a time frame renders our assessment of weight loss success less accurate.

Another major limitation is the convenience sampling method, as college women have very limited generalizability. In fact, 65% of our sample reported body weight and BMIs that were within the normal weight range and yet, also reported trying to lose weight. Given that perceived overweight is often associated with greater disordered eating (Haynes et al., 2018), it is unfortunate that we did not include any indicators of disordered eating. We recommend their inclusion in future studies. A study that includes men would also be helpful. As noted in previous research (Wayment, 1992), males and females may differ in their self-evaluation processes, which may be especially prevalent in the weight loss domain (see Elder, 2012). Another limitation is the cross-sectional design, which precludes any conclusions regarding causality. Longitudinal studies are needed to understand the consequences of specific self-evaluation strategies on multiple motives.

A final limitation relates to validity. Although respondents provided examples of the types of self-evaluative information they used, we do not have good information about college students’ real-time exposure to the actual information they may use for self-evaluation. For example, the low reported use of downward social comparison information may be because women have less access to weight-relevant information about those who are not as successful in their weight loss. Given the idealized framing associated with social media posts, there may also be less downward social comparison information available for most normal sized individuals (Betz et al., 2019). To address this limitation, lab studies employing behavioral measures (e.g., eye-tracking) or field studies using experience sampling methods (cf., Myers et al., 2012; Fardouly et al., 2017) could provide more informative insight about information use preferences in real time.

## Conclusion and Implications

When it comes to the role of psychological processing related to weight loss in college women, self-evaluation is but one possible contributor, mostly in the context of self-monitoring (Kanfer, 1991; Burke et al., 2011). The research presented here examined self-evaluation strategies in a sample of college women, two-thirds of whom, although they reported weight and weight loss goals representative of college samples used in other studies (Anderson et al., 2003; Herring et al., 2014; Vargas et al., 2014; Zeigler-Hill and Noser, 2015; Dakanalis et al., 2016), reported body weights and BMIs within the normal range. However, for college women, even the perception of being overweight (including inaccurate body perceptions) is associated with weight loss goals and practices (Shamaley-Kornatz et al., 2007; Haynes et al., 2018). Thus, one practical implication is to design educational interventions that help women to understand the consequences of the comparisons for setting weight loss goals, monitoring weight loss progress, and maintaining motivation. In some cases, women can be encouraged to making non-weight-related comparisons to meet self-enhancement needs (van den Berg and Thompson, 2007).

For example, educational materials could be produced that encourage the use of objective information for the articulation of a goal (accuracy motive), the selective use of upward social comparison information and objective information to monitor goal progress (self-improvement motive), and the use of objective and downward social comparison) to maintain one’s motivation (self-enhancement). Although not covered in this study, personal forms of information, including feedback from others, are also extremely relevant to these processes (Wayment and Taylor, 1995). Our hope is that these results can contribute to any ongoing effort to raise students’ awareness of how self-evaluative information can be helpful or unhelpful to the setting, monitoring, and maintaining weight-related goal pursuits.

## Data Availability Statement

The datasets generated for this study are available on request to the corresponding author.

## Ethics Statement

The studies involving human participants were reviewed and approved by Northern Arizona University Institutional Research Board. The patients/participants provided their written informed consent to participate in this study.

## Author Contributions

HW and KC designed the study. KC collected the data. HW and BE took the lead on the current manuscript. KC collected the data for this study was part of the thesis work, supervised by HW and BE.

## Conflict of Interest

The authors declare that the research was conducted in the absence of any commercial or financial relationships that could be construed as a potential conflict of interest.

## Footnotes

1 The original Wayment and Taylor (1995) framework also included the use of personal sources of self-evaluative information (personal standards, past positive information, past negative information, ideal future selves, and feared selves).

2 Fifty-one participants were removed from the sample due to satisficing, defined as participants’ tendency to exert minimal effort when participating in a study (Barge and Gehlbach, 2012; Zhang, 2013). Four satisficing metrics were computed: (1) rushing, (2) skipping, (3) straightlining, and (4) early termination. A rushing score was computed by creating a seconds-per-item rate (SPI; the number of seconds participant spent on the survey divided by the number of items completed; Barge and Gehlbach, 2012). A skipping score was computed by dividing the number of questions that were left blank by the total number of questions. The higher the skipping score (i.e., a higher percentage), the more questions skipped. A straightlining score was computed by identifying banks of items that had a standard deviation of 0, indicating no variation across a subset of five or more items (Barge and Gehlbach, 2012). Finally, the number of participants who terminated the survey early were determined. Five respondents were coded positive (“1”) for early termination.

3 One goal of this study was to examine if average rates of information use might differ between those reported by Wayment in 1992 and what we found by studying college students in 2018. In the original 1992 study, the integrative model of self-evaluation processes was examined in two domains (academic performance and social life) in two samples of college students (*N* = 116, *N* = 470). The frequency ratings for information use in the academic domain were as follows: objective (study 1: 5.63, study 2: 5.39), upward social comparison (5.30, 4.44), lateral social comparison (4.40, 4.48), and downward social comparison (3.25, 3.38). The frequency ratings for information use in the social domain were as follows: objective (4.36, 4.72) upward social comparison (4.31, 4.98), lateral social comparison (4.01, 4.51), and downward social comparison (3.31, 3.52). Means from the current study (as reported in Table 1) are very similar to those reported in the 1992 study (objective: 5.04, upward social comparison: 5.28, lateral social comparison: 4.41, downward social comparison: 3.58).
